# A novel method based on selective laser sintering for preparing high-performance carbon fibres/polyamide12/epoxy ternary composites

**DOI:** 10.1038/srep33780

**Published:** 2016-09-21

**Authors:** Wei Zhu, Chunze Yan, Yunsong Shi, Shifeng Wen, Jie Liu, Qingsong Wei, Yusheng Shi

**Affiliations:** 1 State key Laboratory of Materials Processing and Die & Mould Technology, School of Materials Science and Engineering, Huazhong University of Science and Technology, Wuhan 430074, China; 2 Guangdong Silver Age Sci & Tech Co. Ltd, Dongguan 523927, China

## Abstract

A novel method based on selective laser sintering (SLS) process is proposed for the first time to prepare complex and high-performance carbon fibres/polyamide12/epoxy (CF/PA12/EP) ternary composites. The procedures are briefly described as follows: prepare polyamide12 (PA12) coated carbon fibre (CF) composite powder; build porous green parts by SLS; infiltrate the green parts with high-performance thermosetting epoxy (EP) resin; and finally cure the resin at high temperature. The obtained composites are a ternary composite system consisting of the matrix of novolac EP resin, the reinforcement of CFs and the transition thin layer of PA12 with a thickness of 595 nm. The SEM images and micro-CT analysis prove that the ternary system is a three-dimensional co-continuous structure and the reinforcement of CFs are well dispersed in the matrix of EP with the volume fraction of 31%. Mechanical tests show that the composites fabricated by this method yield an ultimate tensile strength of 101.03 MPa and a flexural strength of 153.43 MPa, which are higher than those of most of the previously reported SLS materials. Therefore, the process proposed in this paper shows great potential for manufacturing complex, lightweight and high-performance CF reinforced composite components in aerospace, automotive industries and other areas.

Carbon fibre reinforced polymer (CFRP) composites are highly promising lightweight materials and have been widely used for various applications in military, automotive and aerospace industries^1^. Compared with continuous fibre composites, short fibre reinforced polymers (SFRP) combine easier processability with low manufacturing cost^2^. Therefore, in recent years the use of SFRP composites grows rapidly in many engineering applications. SFRP composites, depending on their matrix materials, can be generally classified into two categories: thermoplastic and thermosetting composites. In terms of commercial application, thermosetting composite parts nearly dominate the composite market owing to their greater thermal and dimensional stability, better rigidity, and higher electrical, chemical and solvent resistance^3^. Nowadays, there are several processing methods available for making short carbon fibre reinforced thermosetting composites, mainly including compression molding and injection molding using sheet molding compounds (SMCs) and bulk molding compounds (BMCs) as raw materials. Most of these techniques involve a molding process, which exhibits high production efficiency and good product accuracy, but unfortunately suffers from a long preparation period and high production cost^3^. Additionally, they are also facing a great challenge in manufacturing parts with high complexity and customized geometries.

Selective laser sintering (SLS), a powder bed fusion additive manufacturing (AM) process, is able to fabricate three dimensional (3D) objects by adding powdered materials layer-by-layer according to computer-aided design (CAD) models. Consequently, it has the capability of building components with complex structures without the need of any tooling^4^,^5^. SLS has already been introduced to make polymer composites for the purpose of improving the capability of SLS parts, thus extending their application as the essential components of the automotive or aeronautical sector^6^. Polyamide (PA), such as PA12 is the most widely used polymer in the current market of SLS materials so far^5^, and abundant investigations have been conducted on the preparation of various PA-based composites for SLS, such as PA/silicon carbide^7^, PA/glass bead^8^, PA/aluminum^9^, PA/carbon nanotube^10^ and PA/carbon nanofibre^11^. Besides PA, some other polymer matrix composites, for instance polycapralactone (PCL)/hydroxyapatite (HA)^12^, PEEK/HA^13^, polyethylene (PE)/HA^14^, and polystyrene (PS)/nano-Al_2_O_3_^15^ were also developed for SLS. In particular, recently, some researchers prepared carbon fibre (CF) reinforced PA (CF/PA) composite powders for SLS^16^, and revealed that the incorporation of CF could largely enhance the mechanical strength of SLS-produced PA parts^17^. In addition, CF/PA composite powders are commercially available for SLS such as Carbonmide^TM^ (EOS, Germany)^18^, Duraform HST^TM^ (3D systems, USA)^19^ and Windform XT^TM^ (CRP Technology, Italy)^20^. However, as discussed above, the majority of polymer composites applied in SLS are based on thermoplastics, and thus suffering from low strength and poor thermal resistance. Much less attention has been paid to the development of high-performance thermosetting composite, especially CF reinforced thermosetting composites, through the SLS process.

Infiltrating the SLS parts with epoxy resin was used as a post-process to make polymer blend as to enhance the mechanical strength of the polymer matrix, such as polycarbonate^21^ and polystyrene^22^, but the result shows limited benefit. Recently, some efforts were made to prepare CF reinforced thermosetting composite by SLS. Bourell *et al.*^23^ and Guo *et al.*^24^ combined the SLS process, furnace carbonization and infiltration of epoxy resin to build graphite-based fuel cell bipolar plates, and CFs were employed to improve the strength of the green parts. The results show that the flexural strength of finished parts increased from 35 MPa to almost 50 MPa through CF additions. When the CF content was 25 vol. %, the flexural strength was almost 50 MPa, which was 1.5 times of the measured flexural strength without CFs. However, the strengths of these thermosetting-based composites mentioned above are still very low and need to be further upgraded for the usage of structural components.

In this work, a novel process based on SLS has been proposed for making high-performance CF reinforced thermosetting resin composites. The feasibility of this method was verified and every single preparation procedure was optimized. The microstructure, mechanical properties and reinforcement mechanisms of the CF reinforced thermosetting resin composites were investigated. The results showed that the prepared composites have a three dimensional co-continuous carbon fibres/PA12/epoxy resin (CF/PA12/EP) ternary structure, presenting the higher tensile and flexural strengths than most of the previously reported SLS materials.

## Results and Discussions

### Process for making CF reinforced thermosetting composite

**Figure 1:**
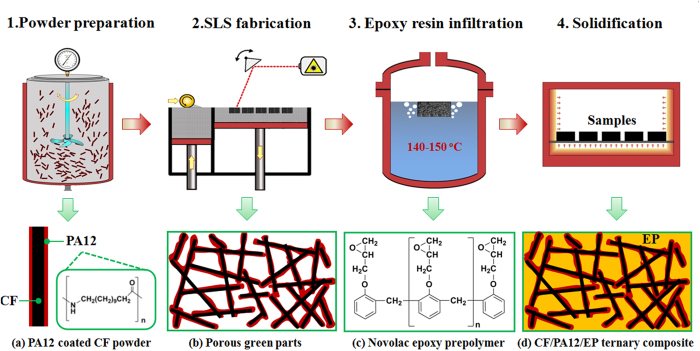
Procedures for the production of CF reinforced thermosetting composite components.

Figure 1 shows the procedures of the process for making CF reinforced thermosetting composites. Firstly, the PA12/CF composite powder for SLS was prepared by coating a thin layer of PA12 on the surfaces of the CFs; secondly, SLS process was used to build green parts with inherent pores; thirdly, the green parts were infiltrated with a high-performance novolac epoxy resin under the condition of high temperature and negative pressure; finally, the composite parts were cured following a three-step curing schedule to obtain a CF/PA12/EP ternary composite.

**Figure 2:**
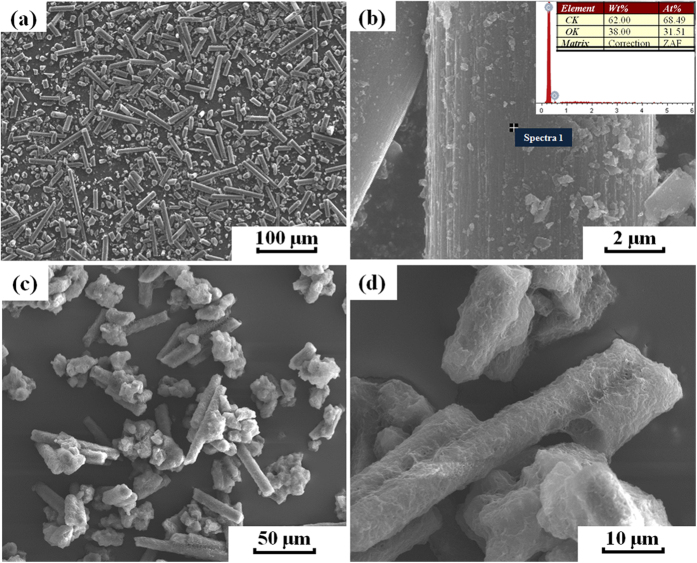
SEM images of (**a,b**) the surface-treated CF powder and (**c,d**) the PA12/CF composite powder.

Figure 2 shows the SEM micrographs of the acid treated CFs and the PA12/CF composite powder. As shown in Fig. 2a, the CF powder is composed of fibres with various lengths. The fibre length distribution is investigated by using Image J^®^ analyzing program, showing that the ultimate lengths are in the range from 1 to 150 μm and the number average fibre length is found to be 55.12 μm. A higher magnification SEM image in Fig. 2b shows that the acid treated CFs have rough surfaces with tiny milled particles adsorbed on it, which is beneficial to the improvement of the interfacial bonding between CFs and PA12. The inset EDX spectrum in the Fig. 2b illustrates the chemical composition on the surface of CF. It is found that the surface contains of C and O (except H, which cannot be detected by EDX) with the content of 62 wt% and 38 wt%, respectively, indicating that some oxygen containing groups are formed on the surfaces which can increase the reactivity with the PA12 layer. Figure 2c reveals that the PA12/CF composite powder is irregularly shaped and remains the original fibre-like morphology. A thin layer of PA12 polymer is uniformly coated on the surfaces of CFs with no fibre surfaces exposed, as shown in Fig. 2d with a higher magnification. The average thickness of this thin PA12 layer is measured to be 595 nm according to the differences between the average diameters of the CFs before and after being coated with PA12. Also, it can be seen that some isometric particle clusters are formed due to the very short fibres and the small fragments, which are favorable to the powder distribution during the SLS process.

**Figure 3:**
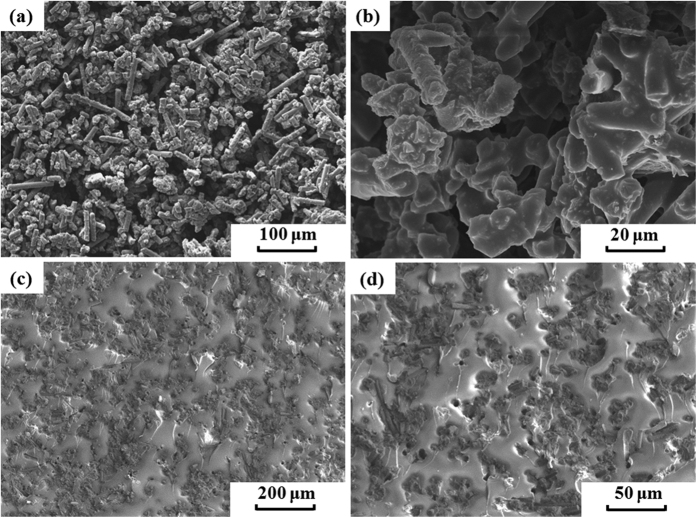
SEM images of (**a,b**) the surfaces of the green parts and (**c,d**) cross-sections of CF/PA12/EP ternary composite.

Figure 3 shows the cross-sections of the porous green parts built by SLS and the final composite after infiltration. From Fig. 3a,b, it can be seen that the PA12 polymer binder is fully melted, and connects the CFs to a self-supported but highly porous structure. The pores inside the structure are three dimensional interconnected owing to intrinsic loose packing of the powder bed, which can be filled up with the liquid epoxy resin through the subsequent infiltration. As shown in Fig. 3c,d, it is clearly evident that the matrix of EP and the reinforcement of CFs interpenetrate into each other, and therefore a three-dimensional co-continuous structure is obtained^25^.

Through the manufacturing process shown in Fig. 1, the CF/PA12/EP ternary composite is finally formed. The dispersed CFs are the reinforcement and the EP serves as the matrix in the system. The thin PA12 polymer layer coating on the surface of CFs serves two purposes: (1) serving as a binder to connect the discrete CFs into a porous CF preform, under the laser irradiation during the SLS process; (2) acting as an intermediate layer to increase the chemical interaction and wettability between CF and EP matrix^26^,^27^. The relative content of PA12 in the starting composite powder determined the initial strength and porosity of the SLS green parts. The more the binder, the higher strength and lower porosity the green parts will have, and finally the less epoxy resin will infiltrate into the composites parts. In this study, the volume fraction of PA12 in the starting PA12/CF composite powder is 25%, which is the optimal content to maximize the porosity under the premise of sufficient strength for post handling.

**Figure 4:**
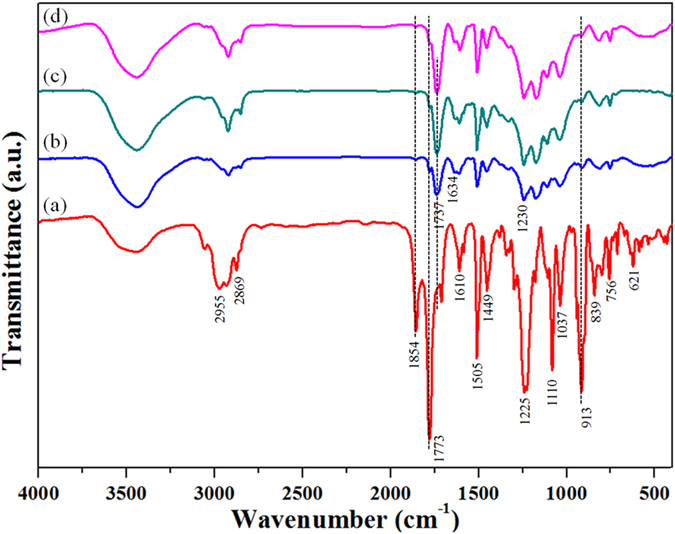
FTIR spectra of the CF/PA12/EP ternary composite (**a**) before curing and after curing at the different stages: (**b**) 120 °C/5 h, (**c**) 120 °C/5 h + 150 °C/3 h, and (**d**) curing at 120 °C/5 h + 150 °C/3 h + 200 °C/2 h.

The infiltration process is operated at 150 °C, which requires that the polymer binder can sustain at such a high temperature without parts distortion. Fortunately, PA12 is such a kind of semi-crystalline polymer with the initial melting temperature of about 182 °C (see Fig. S1 and Table S1), which is sufficient to maintain the CF structure. During the curing process, the reaction of epoxides and anhydrides mainly follows an alternate ring-opening copolymerization mechanism that leads to polyester and polyether networks^28^. Figure 4 shows the FTIR spectra of the composite cured at different stages. The assignment of the absorption peaks are as follows^29^,^30^:3700-3300 cm^−1^ to hydroxyl group, the N-H stretching occurs near 3270 cm^−1^ but cannot be clearly discerned due to the broad hydroxyl region, 2955-2869 cm^−1^ to the CH_3_ and CH_2_, 1854 and 1773 cm^−1^ to anhydride C=O, 1737 cm^−1^ to ester linkage C=O, 1634 cm^−1^ to the stretching vibration of C=O in secondary amide group, 1610, 1505 and 1449 cm^−1^ to the to the stretching and deformation aromatic C=C, 1247 cm^−1^ to the aromatic ether (oxygen atom attached to C atom of aromatic ring), 1110 and 1037 cm^−1^ to the deformation of the aromatic C−H, 913 cm^−1^ to epoxide groups, 839 cm^−1^ to the out-of-plane deformation of the aromatic C−H, 756 and 621 cm^−1^ to the out-of-plane deformation of the anhydride C−H and C−C−C. The structural changes during curing process can be identified from Fig. 4. After cured at 120 °C for 5 h (Fig. 4b), the absorption intensity of hydroxyl with the broad spectral feature increased, while the absorption intensity of anhydride C=O at 1854 and 1773 cm^−1^, and the intensity of epoxide group at 913 cm^−1^, decrease greatly. Correspondingly, the absorption intensity of the C=O in the newly formed ester linkage at 1737 cm^−1^ appeared and became stronger. These changes mentioned above can be attributed to the reaction of anhydride with a hydroxyl forming an ester group and a carboxylic acid, which in turn caused the ring-opening of an epoxide group generating an additional hydroxyl moiety. In the second curing stage (150^ ^°C for 3 h), the ring-opening reactions continues (Fig. 4c). Meanwhile, as the curing temperature of 150 °C in this stage is close to the initial melting temperature of PA12, the molecular chains of PA12 become active and have the possibility to intercalate into the formed three dimensional EP networks, thus improving the interfacial adhesion. As the curing reaction goes into the third stage (200 °C for 2 h), the absorption peaks of epoxy group and anhydride nearly disappear (Fig. 4d). In the meantime, because the curing temperature in this stage is much higher than the melting temperature of PA12, the PA12 phase in the composites is fully melted again and flows in the early shaped rigid “EP resin mold”, which can eventually modify the existent morphology of the PA12 in the composites. And in this stage, the molecular chains of PA12 unfold and some chemical reactions can happen between the melted PA12 and the incompletely cured epoxy resin, and which will be discussed in more detail in the next section. The performance of the composites relies largely on the matrix resin. The novolac epoxy resin used in this study can provide good strengths and chemical resistance at high temperature^31^, but the mechanical properties of the final composite parts can be tailored for different purposes by varying the different types of thermosetting resins that are used.

### Microstructure characterization by micro-CT

**Figure 5:**
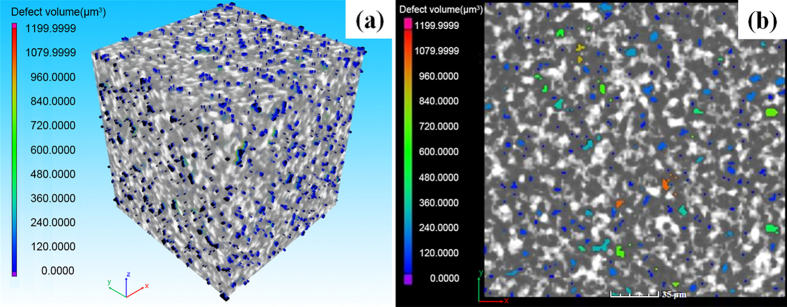
Micro-CT (**a**) visualization of the volume and (**b**) segmented pore distribution of the CF/PA12/EP ternary composites. The white represents CFs, the grey shows the matrix EP and the colour ranged from blue to red denotes the pores with different volumes. It shows that the CFs are uniformly dispersed in the matrix, and the pores are distributed inside the EP domains or on the border of the CF and EP domain.

Micro-CT is a non-destructive method that enables the users to reconstruct a series of 2D projection images into spatial configurations, and to define the internal structure of the composite parts^32^,^33^. The radiography is based on the absorption property of the sample and the image contrast is generated by the variation of density in a material. The density gaps between CFs, EP and the air yield good contrast in the 3D perspective image produced by micro-CT, as shown in Fig. 5a. In the cross-section (Fig. 5b), the CFs are uniformly dispersed in the matrix (see Supplementary Fig. S2 online to visualize the fibre distribution and orientation). Although the fibre diameter is 7~8 μm, single fibre cannot be distinguished even using the best resolution of 1.79 μm per pixel. This may be attributed to the thin layers of PA12 coated on the CF surfaces and the small distances between the CFs, which are less than 1.79 μm and make it impossible to detect single fibre. Similar problem was also found in the previous literature^34^. It also can be seen that the pores are mainly distributed: (1) inside the EP matrix domains. These pores might be formed during the solidification shrinkage of EP resin during the curing process; (2) on the boundaries of the EP and CF domains, which is because the remelted PA12 polymer fills the voids between the CFs during the third curing stage, thus resulting in some small pores on the boundaries. Micro-CT is also a quantitative method that allows quantitative determination of the pore size distribution and phase content within a particular volume. Figure S3 illustrates the average pore size distribution. Nearly 60% of the pores are less than or equal to the minimum resolution of 1.79 μm, and more than 90% of the pores are found to be smaller than 10 μm. The average pore fraction is 4.93%, and the phase fraction of CFs is 31%.

### Mechanical properties

The mechanical properties of CF reinforced composites are largely affected by the volume fraction of fibres, fibre length distribution and the interfacial shear strength between fibres and matrix^35^. This paper establishes a modified Kelly-Tyson model^35^,^36^,^37^ for predicting the tensile strength of the CF/PA12/EP ternary composites, taking into account the effects of fibre lengths as well as fibre-matrix interface. As to a ductile matrix and large strains in the composites, a linear increase of tensile stress in the fibre with distance from the ends can be assumed. The critical fibre length *l*_*c*_ is the theoretical length that achieves sufficient stress transfer between the fibre and matrix to cause failure, which can be expressed by the following equation:


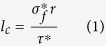


where 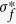 is the fibre strength and *τ*^∗^ is the interfacial shear strength, *r* is radius of the fibre. The critical fibre length is estimated to be 204 μm, using the 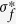 = 3500 MPa, *r* = 3.5 μm, *τ*^∗^ = 60 MPa which are typical values for CF reinforced composites^35^. The reinforcing effect of the fibres increases with their lengths, which are supposed to be exceeding *l*_*c*_ as much as possible to obtain high strength. Nevertheless, the length of the CFs used in this work could not be so long as to interfere with the processability, e.g. distribution and sinterability of the composite powder in the SLS process. Hence, the CFs used in this study are shorter than *l*_*c*_. In this case, the composite strength can be given by^35^,^36^,^37^:


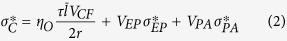


where 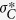 is the ultimate tensile strength of the composite; *η*_*O*_ represents the factor with respect to the fibre orientation; *τ* is the average interfacial shear strength between CFs and EP matrix; 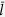 and *r* denote the average length and radius of fibres respectively; *V*_*CF*_, *V*_*EP*_ and *V*_*PA*_ represent the volume fractions of CFs, EP matrix and PA12 in the final composite parts respectively; 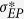 and 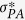 are the ultimate tensile strengths of the pure block of EP matrix and PA12, respectively.

The following values are assumed for the samples: *η*_*O*_ = 0.375, for the fibres are in-plane randomly distributed^35^,^38^; *τ = *60 MPa, 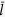 = 55.12 μm, *r* = 3.5 μm; 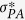 = 46 MPa^39^, 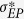 = 85 MPa according to the specification of the manufacturer; *V*_*CF*_ = 31% obtained from the result of mico-CT, and *V*_*PA*_ = 7.75% calculated from the initial volume ratio of PA12 and CFs (1: 3) in the composite powder, and then *V*_*EP*_ counting for the remaining volume of 61.25% regardless of the porosity. Therefore, a theoretical ultimate tensile strength for the ternary composite built by this method is calculated to be 110.55 MPa, using Eq. 2.

The tensile and flexural properties of the final CF/PA12/EP ternary composite parts are experimentally tested, and the typical tensile and flexural stress-strain curves of the CF/PA12/EP ternary composites are shown in Fig. S4. The ultimate tensile strength is determined to be 101.03 MPa, which is slightly lower than but very closed to the predicted value. The flexural strength is also evaluated to be 153.43 MPa.

As a benchmark, we have compared the mechanical properties of some polymer-based materials built by the SLS process reported in the previous literature alongside our results in Table 1. Polyaryletherketone family polymers, such as polyether ether ketone (PEEK) and polyether ketone (PEK), are top-ranked SLS materials by far in terms of mechanical performance. Nevertheless, it can be found from the data listed in Table 1 that the CF/PA12/EP ternary composites produced in this work exhibit higher tensile strength and flexural strengths than most of the reported SLS materials, including PA12, CF/PA12 composites, PEK and PEEK. Hence, our composites are competitive with the present SLS materials. The process proposed in this paper shows great potential for manufacturing complex, lightweight and high-performance CF reinforced composite components in aerospace, automotive industries and other areas.

**Figure 6:**
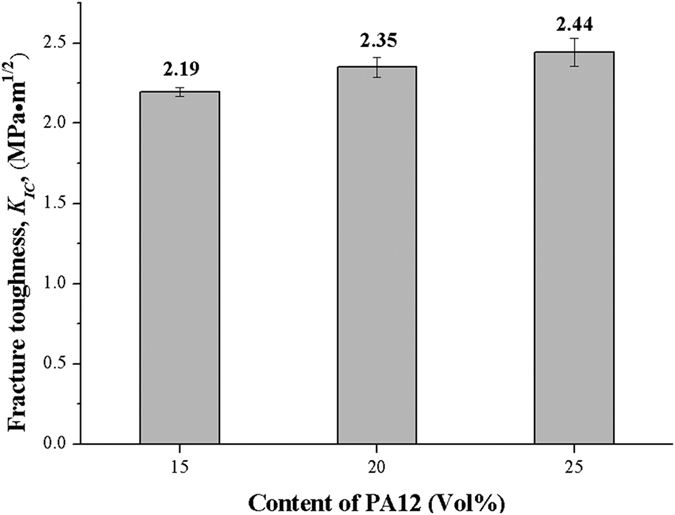
Fracture toughness of the CF/PA12/EP ternary composite with respect to the PA12 content.

The effect of PA12 content on the fracture toughness of the CF/PA12/EP ternary composites is shown in Fig. 6. As the PA12 content was increased, the *K*_*IC*_ value gradually increased. When the PA12 content was 25 Vol%, the fracture toughness was increased by 11% compare to the content of 15 Vol% on the average. It was concluded that with the addition of PA12 phase, the fracture toughness can be improved.

### Reinforcement mechanism analysis

**Figure 7:**
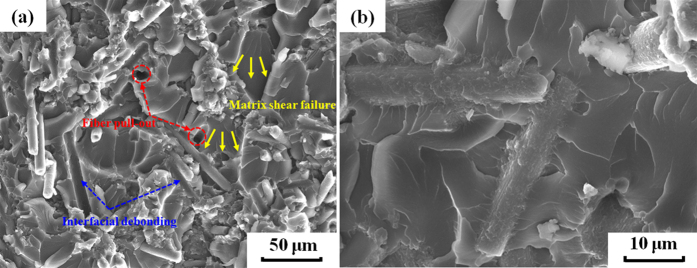
Tensile fracture surfaces of the CF/PA12/EP ternary composite at magnification of (**a**) 500×, (**b**) 2000×.

The SEM micrographs of tensile fracture surfaces of the CF/PA12/EP ternary composites are shown in Fig. 7. The composites exhibit a typical brittle failure behavior, but the whole fracture surface is characterized by a much rough morphology with shear deformation, as shown in Fig. 7a. The CFs, PA12 and EP can be clearly distinguished from the fracture surfaces. It can be observed that the deformation and cracks of the EP matrix propagate in different directions (indicating by arrows), because the crack expansion in the brittle EP matrix is blocked by the network-like CF/PA12-rich domain and forced to change their tracks, which significantly contributes to the improvement of the fracture toughness and strength of the composites. The failure mechanisms in short fibre reinforced composites include fibre breakage, fibre pullout, interfacial debonding and matrix failure^40^. More specifically for the composites in this work, the last three kinds of mechanisms except for fibre breakage can be observed on failure surfaces, as denoted in Fig. 7a. Because the CFs used in this study are much shorter than the critical fibre length, the maximum load transferred from the matrix to the fibre is not sufficient to lead to a fibre breakage.

**Figure 8:**
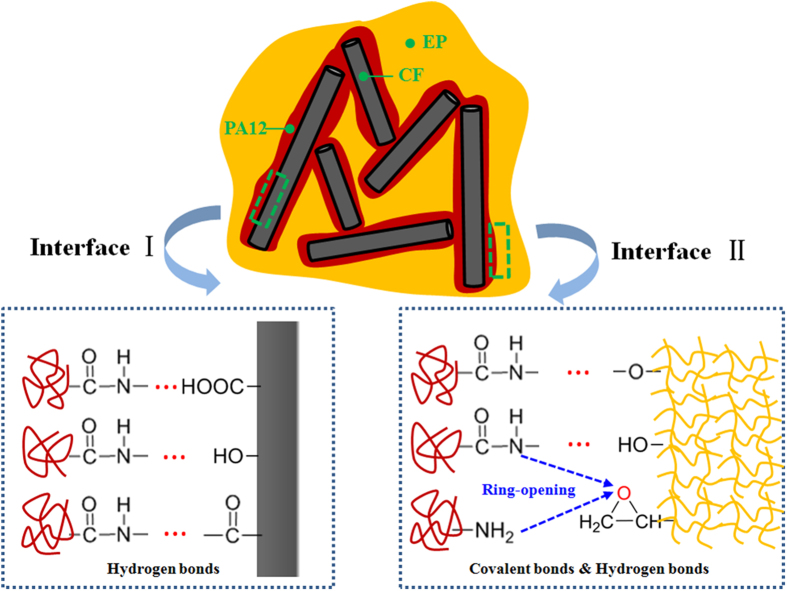
Sketches of the composite and the chemical reactions at the interfaces.

Compared to most of the previously reported SLS materials, the CF/PA12/EP ternary composites produced in this work possess higher tensile and flexural strengths, which can be attributed to: (1) uniformly distributed reinforcing CFs and (2) good interfacial adhesion between CFs and EP matrix through the modification of the 595 nm PA12 intermediate layer, as shown in Fig. 7b. There are several mechanisms for increasing the fibre-matrix bonding, involving mechanical interlocking, adsorption interaction, electrostatic attraction and molecular chain entanglement^41^. As far as the composites in this study are concerned, the mechanical interlocking could be an important factor. As shown in Fig. 2, the surfaces of coated CFs are relatively rough, indicating that the surface area of the CFs is increased and thus the more mechanical interlocking sites are formed. In addition, the enhancement of the surface roughness reduces the contact angle between the CFs and matrix and hence increases the wettability^41^ as well. Another important reason for the good interfacial adhesion is the chemical interaction. There are two interfaces in this ternary composite system, as shown in Fig. 8. One is in between CF and PA12. It is reported that the acid treatment could increase the concentration of functional groups, e.g. hydroxyl (−OH), carbonyl (−C=O) and carboxyl (−COOH) groups. These oxygen containing functional groups (hydrogen bond acceptor) on the CF surface have a propensity to form hydrogen bonds with amide groups (hydrogen bond donor) in PA12^42^, especially when the original hydrogen bond between the N−H and C=O groups of adjacent chains inside the PA12 is broken during the third curing stage. The other interface is in between the PA12 intermediate layer and the EP matrix, which plays a critical role in transferring load between the reinforcing CFs and matrix. In the third curing stage (200 °C for 2 h), the PA12 is remelted in liquid form and the EP resin has not been completely cured; therefore, abundant hydrogen bonds can form in the same way between amide groups and the products of epoxide ring-opening, such as hydroxyl and ether oxygen group (−O−). Moreover, some covalent bonds are fashioned through the interaction between the unopened epoxide ring in the EP and the terminal amino groups (−NH_2_) or amide groups (CONH−) in PA12^43^,^44^. Therefore, these chemical reactions that take place at the interface can greatly improve the interfacial bonding strength.

The CF/PA12/EP ternary composite parts with complex structures have been manufactured by this method. These parts are strong enough to be used as structural components after post polishing processing. Some of them are shown in Fig. S5.

## Conclusions

In summary, the high-performance CF reinforced thermosetting composites with a CF/PA12/EP ternary structure were successfully prepared by a novel method based on SLS process in this work.

(1) From the SEM observation and micro-CT analysis, it is evident that the ternary system forms a three-dimensional co-continuous structure, and the CF reinforcement can be well dispersed into the EP matrix with volume fraction of 31%. The PA12 phase not only serves as a binder to connect CFs to generate complex geometries in the SLS process, but also a transition thin layer with a thickness of 595 nm to improve the interfacial bonding.

(2) Mechanical tests shows that the composites fabricated by this method yield an ultimate tensile strength of 101.03 MPa and a flexural strength of 153.43 MPa, and are stronger than most of the previously reported SLS materials.

(3) A modified Kelly-Tyson model is established for the prediction of tensile strength of the CF/PA12/EP ternary composites. The predicted ultimate tensile strength for the ternary composite is 110.55 MPa, which is slightly higher than but very closed to the measured experimental value.

(4) There are two main mechanisms for good interfacial adhesion between the CFs, PA12 and EP matrix. One is the enhanced mechanical interlocking owing to the surface modification of the thin layer of PA12. The other is the chemical interaction to form hydrogen bonds and covalent bonds on the interfaces.

Therefore, the process based on additive manufacturing proposed in this paper work shows promising potential method to manufacture large, complex and lightweight structural components in aerospace, automotive industries and other areas. Moreover, it is also a flexible method because the properties of the composites can be readily tailored by altering different infiltration resins.

## Methods

### Raw materials

The CF powder used in this study was supplied by Jilin Fangda Jiangcheng Carbon Fibre Co., Ltd., China. It has a density of 1.76 g/cm^3^. The PA12 pellets with a density of 1.01 g/cm^3^ were purchased from Degussa Co., Germany. The thermosetting resin used for infiltration in this work was an epoxy resin (EP) including an epoxy prepolymer and curing agent. The novolac epoxy prepolymer (F51) with the epoxy value of 0.53~0.59 and density of 1.22 g/cm^3^ was provided by Jiangsu Sanmu Group Corporation, China, showing a transparent, yellowish, and sticky liquid. The curing agent was methylnadic anhydride (MNA) accelerated with a trace of 2, 4, 6-tri-(dimethyl aminomethyl) phenol (DMP-30^TM^). The nitric acid with a concentration of 67% was obtained from CNPC Jilin Chemical Co. Ltd., China.

### Preparation of the PA12 coated CF (PA12/CF) composite powder

The PA12/CF composite powder (with a volume ratio of 1:3) for SLS was prepared by a dissolution-precipitation method as detailed in our previous work^16^. The dissolution-precipitation process can be briefly described as follows: firstly, the CFs were surface-treated by the concentrated nitric acid at 60 °C for 2.5 h under ultrasonic oscillation. Secondly, the PA12 pellets, treated carbon fibres and ethanol solvent were added into a sealed reactor, and then the mixture was vigorously stirred during heating to 145^ ^°C, at which the PA12 pellets dissolved thoroughly to form a homogeneous CF suspension. After maintaining this temperature for 2–3 h, the suspension was gradually cooled down to ambient temperature, and the PA12 crystallized taking the CFs as heterogeneous nuclei. Finally, the PA12/CF composite powder was obtained after distilling out solvent, vacuum drying and ball milling.

### Fabrication of green parts through SLS

The sintering experiments were performed on the HK S320^TM^ SLS machine (Wuhan Huake 3D Technology Co. Ltd., China). The SLS system was equipped with a power continuously adjustable CO_2_ laser with a wavelength and beam diameter of 10.6 μm and 200 μm, respectively. The processing parameters were optimized as follows: the laser power was 12 W, the scanning speed was 2000 mm/s, the powder layer thickness was 0.15 mm, the scan spacing was 0.15 mm and the part bed temperature was set as 145 °C.

### Infiltration and solidification of EP to make final composite parts

Prior to infiltration, the epoxy resin was prepared as follows: the novolac epoxy prepolymer was first heated to 150 °C to decrease its viscosity, then the prepolymer was blended with the hardener MNA and accelerator DMP-30 with a weight ratio of 100: 91: 0.15, and the mixture was mechanically stirred until a transparent liquid was obtained. In the infiltration process, the SLS-fabricated porous green parts were immersed into the liquid resin, keeping their top surface exposed to air. The whole process was conducted in a vacuum drying oven at a constant temperature of 150 °C with a negative pressure to ensure that the porous green parts were thoroughly saturated. After cleaning the excess resin, the final composite parts were cured in the oven. A three-step curing schedule was used: 5 h at 120 °C, then 3 h at 150 °C, afterwards 2 h at 200 °C.

### Measurements

The micromorphologies of the powders and composite parts were examined using a field scanning electron microscope (FESEM, JSM-7600F JEOL) and an environmental scanning electron microscopy (ESEM, Quanta 200 FEI). All samples were sputter-coated with platinum to avoid charging. Fourier transform infrared (FTIR, Vertex 70 Bruker) measurements were taken at regular intervals to study the curing process of the composite, using the KBr disk method. The porosity and phase distribution of the prepared composite parts were determined using a microcomputed topography (micro-CT) instrument (dinodo d2, Germany). A rectangular-cut sample for the micro-CT analysis was cut from the tensile test specimen with dimensions of approximately 2 × 2 × 3 mm^3^. Data were collected at 80 kV and 100 μA, computer 3D reconstruction of the parts were made using the software package VGStudio MAX (Volume Graphics GmbH). Mechanical properties of the composites were evaluated using tensile and flexural testing. Tensile samples were finished and tested as per ASTM D638, and three point flexural testing was performed in accordance with ASTM D790, using a universal testing machine (Zwick/Roell Z010, Ulm, Germany) at a crosshead speed of 1 mm/min and 2 mm/min, respectively. Single edge notched beam (SENB) was used to measure the fracture toughness of the composite. Composite specimens with different content of PA12, 15, 20 and 25 Vol %, were fabricated and four notched samples for each content were tested. The ratio of the notch depth to specimen height (*a*/*W*) was in the range of 0.45–0.55. The specimen height was measured by caliper, while the notch depth was measured by optical microscope. Three-point bending tests were carried out at a loading speed of 0.5 mm/min using Zwick/Roell Z010. The span was 32 mm. The fracture toughness was calculated using the Equation (3):


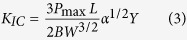


where *P* is the maximum load during three-point bending test, *L* is the bending span, *B* is the specimen width (4 mm), *W* is the specimen height (8 mm), *a* is the notch depth, *α* is the ratio of *a* and W, and *Y* is the calibration factor, which can be calculated by Equation (4):


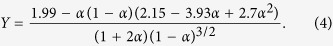


## Additional Information

**How to cite this article**: Zhu, W. *et al.* A novel method based on selective laser sintering for preparing high-performance carbon fibres/polyamide12/epoxy ternary composites. *Sci. Rep.* **6**, 33780; doi: 10.1038/srep33780 (2016).

## Supplementary Material

Supplementary Information

## Figures and Tables

**Table 1:** Comparison of our CF/PA12/EP ternary composites with some polymer-based materials fabricated by SLS.

Sources	Materials	Tensile strength (MPa)	Flexural strength (MPa)
Ref. 45	PEK	~90	—
Refs 5 and 46	PA12	40~48	65
EOS^17^	CF/PA12	72	—
3D Systems^18^	CF/PA12	50.68	—
Ref. 15	CF/PA12	—	~113
This study	CF/PA12/EP	101.03	153.43

## References

[b1] Song W., Gu A., Liang G. & Yuan L. Effect of the surface roughness on interfacial properties of carbon fibers reinforced epoxy resin composites. Applied surface science 257, 4069–4074 (2011).

[b2] Zhang H., Zhang Z. & Friedrich K. Effect of fiber length on the wear resistance of short carbon fiber reinforced epoxy composites. Composites Science and Technology 67, 222–230, doi: 10.1016/j.compscitech.2006.08.001 (2007).

[b3] Mazumdar S. Composites manufacturing: materials, product, and process engineering. (CrC press, 2001).

[b4] Kumar S. & Kruth J.-P. Composites by rapid prototyping technology. Materials & Design 31, 850–856 (2010).

[b5] Goodridge R., Tuck C. & Hague R. Laser sintering of polyamides and other polymers. Progress in Materials Science 57, 229–267 (2012).

[b6] Mario D. M., Rubén P., Fernando O., Jose A. C. & Claudio C. Process for reinforcing SLS parts by epoxy resin. Rapid Prototyping J 21, 322–328, doi: 10.1108/RPJ-08-2013-0079 (2015).

[b7] Gill T. & Hon K. Experimental investigation into the selective laser sintering of silicon carbide polyamide composites. Proceedings of the Institution of Mechanical Engineers, Part B: Journal of Engineering Manufacture 218, 1249–1256 (2004).

[b8] Chung H. & Das S. Processing and properties of glass bead particulate-filled functionally graded Nylon-11 composites produced by selective laser sintering. Materials Science and Engineering: A 437, 226–234 (2006).

[b9] Mazzoli A., Moriconi G. & Pauri M. G. Characterization of an aluminum-filled polyamide powder for applications in selective laser sintering. Materials & Design 28, 993–1000 (2007).

[b10] Salmoria G. V., Paggi R. A., Lago A. & Beal V. E. Microstructural and mechanical characterization of PA12/MWCNTs nanocomposite manufactured by selective laser sintering. Polymer Testing 30, 611–615 (2011).

[b11] Goodridge R. *et al.* Processing of a Polyamide-12/carbon nanofibre composite by laser sintering. Polymer Testing 30, 94–100 (2011).

[b12] Wiria F., Leong K., Chua C. & Liu Y. Poly-ε-caprolactone/hydroxyapatite for tissue engineering scaffold fabrication via selective laser sintering. Acta Biomaterialia 3, 1–12 (2007). 17055789 10.1016/j.actbio.2006.07.008

[b13] Tan K. *et al.* Scaffold development using selective laser sintering of polyetheretherketone–hydroxyapatite biocomposite blends. Biomaterials 24, 3115–3123 (2003). 12895584 10.1016/s0142-9612(03)00131-5

[b14] Hao L., Savalani M., Zhang Y., Tanner K. & Harris R. A. Effects of material morphology and processing conditions on the characteristics of hydroxyapatite and high-density polyethylene biocomposites by selective laser sintering. Proceedings of the Institution of Mechanical Engineers, Part L: Journal of Materials Design and Applications 220, 125–137 (2006).

[b15] Zheng H., Zhang J., Lu S., Wang G. & Xu Z. Effect of core–shell composite particles on the sintering behavior and properties of nano-Al 2 O 3/polystyrene composite prepared by SLS. Materials Letters 60, 1219–1223 (2006).

[b16] Yan C., Hao L., Xu L. & Shi Y. Preparation, characterisation and processing of carbon fibre/polyamide-12 composites for selective laser sintering. Composites Science and Technology 71, 1834–1841 (2011).

[b17] Floersheim R. B., Hou G. & Firestone K. CFPC material characteristics and SLS prototyping process. Rapid Prototyping J 15, 339–345 (2009).

[b18] Material Database of EOS. Available at: http://www.eos.info/material-p. (Accessed: 9th January 2016). (2016).

[b19] Selective Laser Sintering (SLS) Material Properties. Available at: http://www.3dsystems.com/quickparts/prototyping-pre-production/selective-laser-sintering-sls/materials. (Accessed: 9th January 2016). (2016).

[b20] WINDFORM XT 2.0 Technical Sheet. Available at: http://www.windform.com/windform-xt-2-0.html. (Accessed: 9th January 2016). (2016).

[b21] Shi Y., Chen J., Wang Y., Li Z. & Huang S. Study of the selective laser sintering of polycarbonate and postprocess for parts reinforcement. Proceedings of the Institution of Mechanical Engineers Part L-Journal of Materials-Design and Applications 221, 37–42, doi: 10.1243/14644207jmda65 (2007).

[b22] Shi Y. S., Wang Y., Chen J. B. & Huang S. H. Experimental investigation into the selective laser sintering of high-impact polystyrene. Journal of Applied Polymer Science 108, 535–540, doi: 10.1002/App.27686 (2008).

[b23] Bourell D., Leu M.-C., Chakravarthy K., Guo N. & Alayavalli K. Graphite-based indirect laser sintered fuel cell bipolar plates containing carbon fiber additions. CIRP Annals-Manufacturing Technology 60, 275–278 (2011).

[b24] Guo N. & Leu M. C. Effect of different graphite materials on the electrical conductivity and flexural strength of bipolar plates fabricated using selective laser sintering. international journal of hydrogen energy 37, 3558–3566 (2012).

[b25] Ren Z., Jin P., Cao X., Zheng Y. & Zhang J. Mechanical properties and slurry erosion resistance of SiC ceramic foam/epoxy co-continuous phase composite. Composites Science and Technology 107, 129–136 (2015).

[b26] Ma L. *et al.* Effects of bonding types of carbon fibers with branched polyethyleneimine on the interfacial microstructure and mechanical properties of carbon fiber/epoxy resin composites. Composites Science and Technology 117, 289–297 (2015).

[b27] Flores M., Fernández-Francos X., Ferrando F., Ramis X. & Serra À. Efficient impact resistance improvement of epoxy/anhydride thermosets by adding hyperbranched polyesters partially modified with undecenoyl chains. Polymer 53, 5232–5241 (2012).

[b28] Yan C., Shi Y., Yang J. & Liu J. Preparation and selective laser sintering of nylon-12 coated metal powders and post processing. Journal of Materials Processing Technology 209, 5785–5792 (2009).

[b29] Socrates G. Infrared Characteristic Group Frequencies.(Wiley-Interscience, 1994).

[b30] Sadtler. *The Infrared Spectra Atlas of Monomers and Polymers* (Sadtler Research Laboratories, 1980).

[b31] Zhao S., Zhang G., Sun R. & Wong C. Multifunctionalization of novolac epoxy resin and its influence on dielectric, thermal properties, viscoelastic, and aging behavior. Journal of Applied Polymer Science 131 (2014).

[b32] Shen H., Nutt S. & Hull D. Direct observation and measurement of fiber architecture in short fiber-polymer composite foam through micro-CT imaging. Composites Science and Technology 64, 2113–2120 (2004).

[b33] Cosmi F., Bernasconi A. & Sodini N. Phase contrast micro-tomography and morphological analysis of a short carbon fibre reinforced polyamide. Composites Science and Technology 71, 23–30 (2011).

[b34] Schell J., Renggli M., Van Lenthe G., Müller R. & Ermanni P. Micro-computed tomography determination of glass fibre reinforced polymer meso-structure. Composites Science and Technology 66, 2016–2022 (2006).

[b35] Eriksson P. A., Albertsson A. C., Boydell P., Prautzsch G. & Månson J. A. Prediction of mechanical properties of recycled fiberglass reinforced polyamide 66. Polymer composites 17, 830–839 (1996).

[b36] Kelly A. & Tyson a. W. Tensile properties of fibre-reinforced metals: copper/tungsten and copper/molybdenum. Journal of the Mechanics and Physics of Solids 13, 329–350 (1965).

[b37] Ogi K., Nishikawa T., Okano Y. & Taketa I. Mechanical properties of ABS resin reinforced with recycled CFRP. Advanced Composite Materials 16, 181–194 (2007).

[b38] Hull D. & Clyne T. An introduction to composite materials. (Cambridge university press, 1996).

[b39] VESTAMID L Polyamide 12-innovative and reliable. Available at: http://www.vestamid.com/sites/lists/PP-HP/Documents/VESTAMID-L-compounds-characteristics-EN.pdf. (Accessed: 9th January 2016). (2016).

[b40] Avanzini A., Donzella G., Gallina D., Pandini S. & Petrogalli C. Fatigue behavior and cyclic damage of peek short fiber reinforced composites. Composites Part B: Engineering 45, 397–406 (2013).

[b41] Zhang H., Zhang Z. & Breidt C. Comparison of short carbon fibre surface treatments on epoxy composites: I. Enhancement of the mechanical properties. Composites science and technology 64, 2021–2029 (2004).

[b42] Meng H., Sui G. X., Fang P. F. & Yang R. Effects of acid-and diamine-modified MWNTs on the mechanical properties and crystallization behavior of polyamide 6. Polymer 49, 610–620. (2008).

[b43] Yamada K., Haraguchi T. & Kajiyama T. Plasma-graft polymerization of vinyl monomer with an acid amide group onto a surface of carbon fiber and its adhesion to epoxy resin. Journal of applied polymer science 75, 284–290 (2000).

[b44] Tanaka K., Allen S. A. B. & Kohl P. A. Variable frequency microwave curing of amide-epoxy based polymers. Components and Packaging Technologies, IEEE Transactions on 30, 472–477 (2007).

[b45] Ghita O., James E., Trimble R. & Evans K. Physico-chemical behaviour of poly (ether ketone) (PEK) in high temperature laser sintering (HT-LS). Journal of Materials Processing Technology 214, 969–978 (2014).

[b46] Munguia J., Akande S. & Dalgarno K. Compliant flexural behaviour in laser sintered nylon structures: Experimental test and Finite Element Analysis–correlation. Materials & Design 54, 652–659 (2014).

