# Supplementary material for: A novel method based on selective laser sintering for preparing high-performance carbon fibres/polyamide12/epoxy ternary composites
Source: Sci Rep. 2016 Sep 21;6:33780. doi: 10.1038/srep33780 (PMC5030666; doi:10.1038/srep33780)
Supplement: Supplementary Information [file srep33780-s1.pdf]

Supplementary Information

A novel method based on selective laser sintering for preparing high-performance carbon fibres/polyamide12/epoxy ternary composites

Wei Zhu[1], Chunze Yan[1,*], Yunsong Shi[2], Shifeng Wen[1,*], Jie Liu[1], Qingsong Wei[1] & Yusheng Shi[1]

[1] State key Laboratory of Materials Processing and Die & Mould Technology, School of Materials Science and Engineering, Huazhong University of Science and Technology, Wuhan 430074, China

[2] Guangdong Silver Age Sci & Tech Co. Ltd, Dongguan 523927, China

A Diamond differential scanning calorimeter (DSC, Perkin Elmer Instruments Ltd.) was used to evaluate the melting and crystallization characteristics of the PA12 powder made by the dissolution-precipitation process. Samples were heated at a rate of 10°C /min from room temperature to 200°C under the protection of argon gas, and then cooled down at 5°C /min. The result is shown in Figure S1 and Table S1. The degree of crystallinity (X_c) of PA12 powder is calculated according to the following equation:

$$X_c = \frac{\Delta H_m}{\Delta H_m^0} \times 100\%,$$

where ΔH_m is the measured value of melting enthalpy of a sample during the heating procedure, ΔH_m^0 is the melting enthalpy of the 100% crystalline PA12, and ΔH_m^0 is taken equal to 209.2 J/g [1]

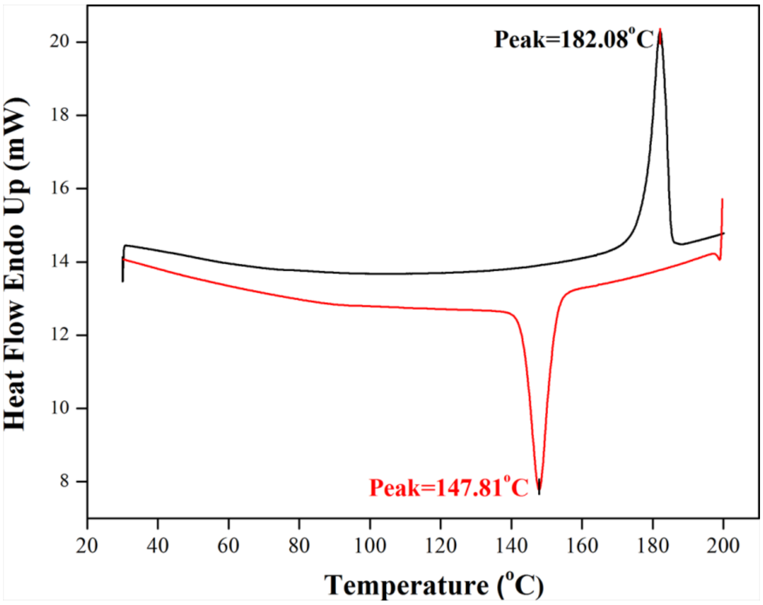

Figure S1. The DSC curve of the pure PA12 powder made by dissolution-precipitation method

Table S1. Melting characteristics of the PA12 powder through the dissolution-precipitation method

	T_{im} (^{o}C)	T_{pm} (^{o}C)	T_{ic} (^{o}C)	T_{pc} (^{o}C)	ΔH_m (J/g)	ΔH_c (J/g)	X_c(%)
PA12 powder	177.58	182.08	152.27	147.81	91.91	-45.39	43.93

Micro-CT: As shown in Fig. S1, the colour bar on the left indicates the range of fibre volume fraction in a segmented area. The cross-section shows a green view on the whole, indicating that the carbon fibres are uniformly dispersed with an average fibre volume fraction of around 31%. The direction of the major axis of the elliptical shape dots in the centre of the segmented square denotes the orientations of the fibres, showing a randomly oriented manner of the carbon fibres.

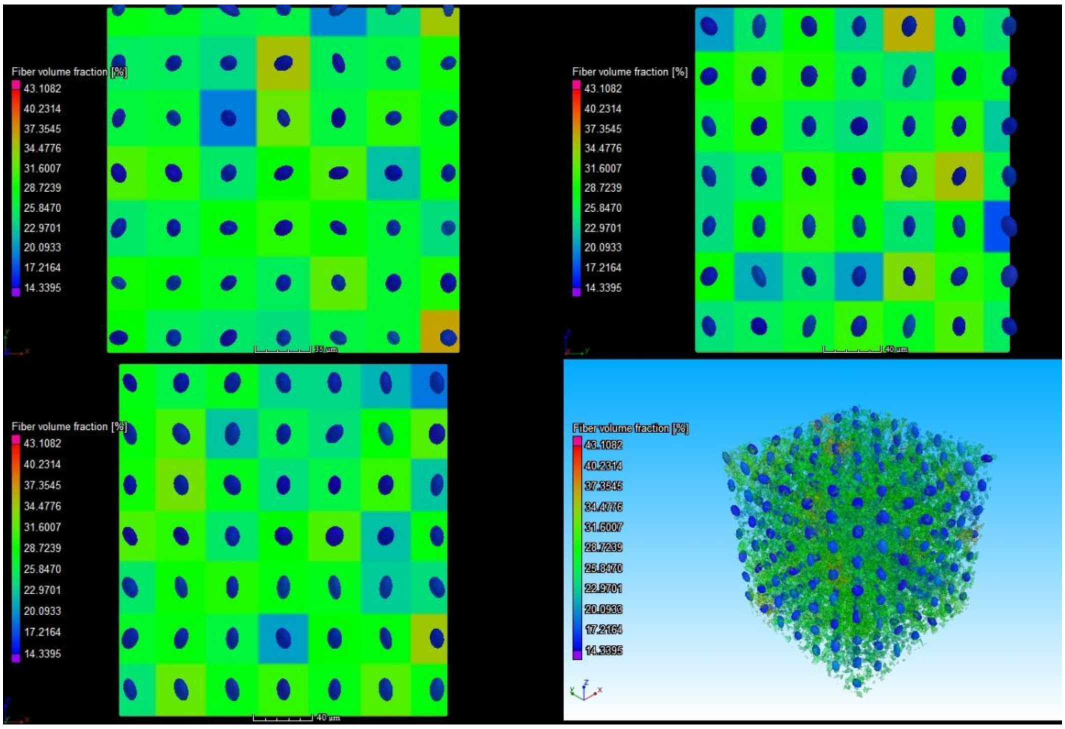

Figure S2. Micro-CT visualization of the fibre distribution and the fibre orientation within a specific volume.

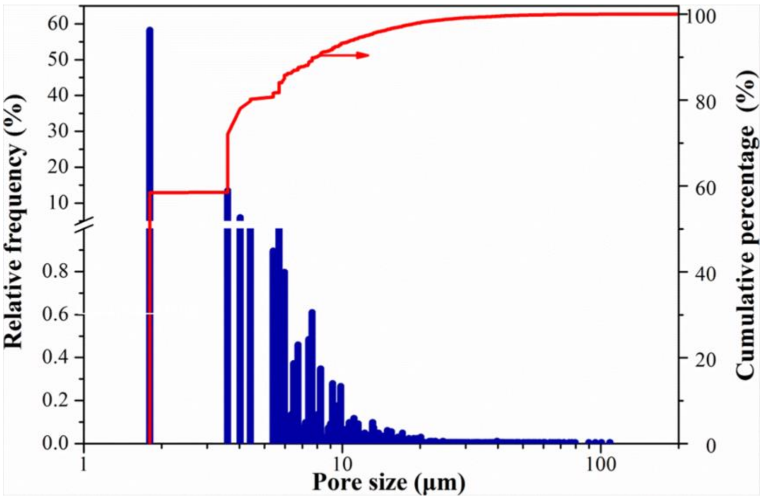

Figure S3. The pore size distribution of the sub-volumes analysed by Micro-CT

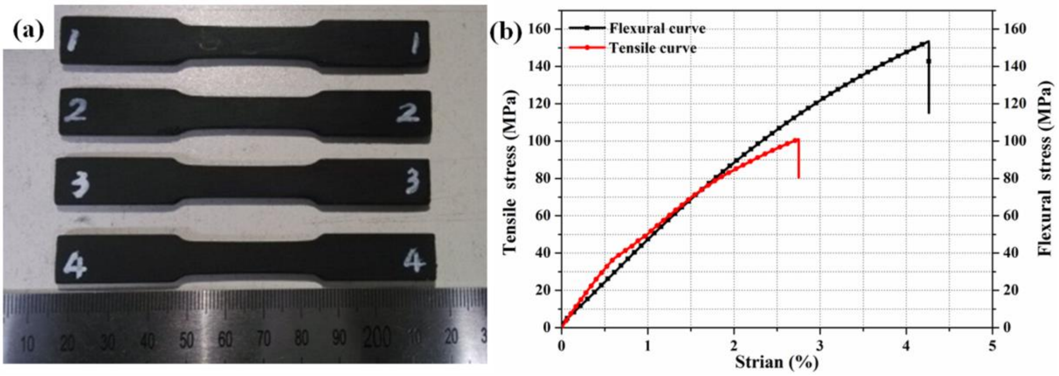

Figure S4. (a) The tensile specimens and (b) typical tensile and flexural stress versus strain curves of the CF/PA12/EP ternary composites.

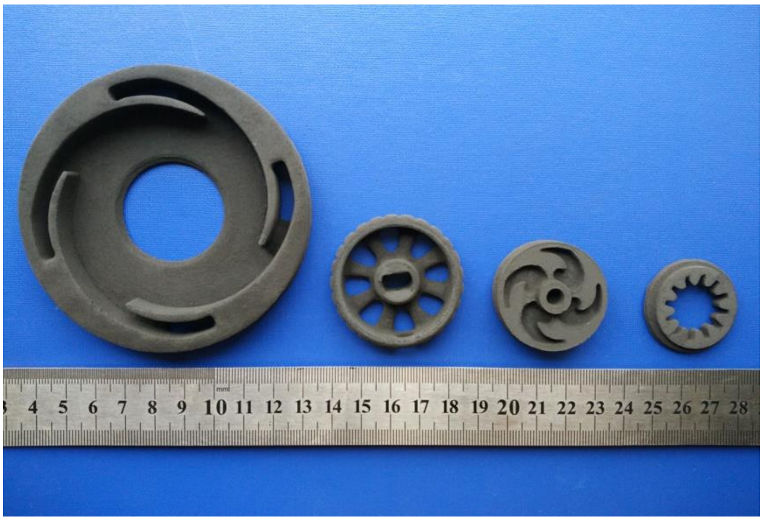

Figure S5. Some CF/PA12/EP ternary composite parts with complex geometries manufactured by the method proposed in this work.

Reference

1. Gogolewski, S., Czerntawska, K., & Gastorek, M. Effect of annealing on thermal properties and crystalline structure of polyamides. Nylon 12 (polylaurolactam). *Colloid and Polymer Science*, **258**, 1130-1136, (1980).
